# Association between non-high-density lipoprotein cholesterol to high-density lipoprotein cholesterol ratio and telomere length: the NHANES 1999–2002

**DOI:** 10.3389/fcvm.2024.1407452

**Published:** 2025-01-06

**Authors:** Mingjie Liu, Chendong Wang, Bai Wei

**Affiliations:** ^1^ Department of Oncology, Liyuan Hospital, Tongji Medical College, Huazhong University of Science and Technology, Wuhan, Hubei, China; ^2^ Hepatic Surgery Center, Tongji Hospital, Tongji Medical College, Huazhong University of Science and Technology, Wuhan, Hubei, China

**Keywords:** NHHR, lipid ratio, telomere length, NHANES database, cross-sectional study

## Abstract

**Background:**

The relationship between non-high-density lipoprotein cholesterol to high-density lipoprotein cholesterol ratio (NHHR) and telomere length (TL) remains unclear. This study aims to investigate their association in a nationally representative US population.

**Methods:**

Data from 6,342 adults aged ≥20 were obtained from the National Health and Nutrition Examination Survey (NHANES) 1999–2002. The NHHR was calculated and categorized into tertiles. TL was measured as the telomere-to-standard reference DNA ratio. Multivariate linear regression and smooth curve fitting were employed to assess the association between NHHR and TL.

**Results:**

The study population (mean age 45.1 ± 0.4 years, 48.9% male) was stratified into NHHR tertiles. Compared with the lowest NHHR tertile, the highest NHHR tertile was associated with adverse inflammatory and cardiometabolic profiles, including elevated white blood cell counts (6.88 ± 0.07–7.54 ± 0.08 × 10^9^/L) and increased prevalence of hypertension (18.81%–25.71%) and diabetes (3.38%–7.17%). An elevated NHHR was significantly associated with a shorter TL (T/S ratio: 1.09 ± 0.02–1.03 ± 0.02; *P* = 0.0005). This association remained significant in partially adjusted models but was attenuated in a fully adjusted model. Significant interactions were observed for age and hypertension status.

**Conclusion:**

This study revealed a linear inverse association between NHHR and TL, suggesting the utility of the NHHR as a novel biomarker for biological aging. Further prospective studies are warranted to validate these findings.

## 1 Introduction

Telomeres, the protective caps at the ends of chromosomes, play a pivotal role in cellular aging and stability. The enzyme telomerase is indispensable for maintaining telomere length (TL) through the addition of telomeric DNA, which is crucial for the normal functioning of cells and the longevity of organisms (1). However, with each cell division, telomeres undergo a process of shortening, which accelerates with age and is linked to various age-related diseases (2). The shortening of TL has been proposed as a biomarker for aging, as it correlates with increased oxidative stress and inflammation, both of which can accelerate telomere attrition (3). A reduction in telomere length in peripheral blood leukocytes has been linked to an elevated risk of cardiovascular disease and certain types of cancer, underscoring its importance in the context of age-related disorders (4). Moreover, telomere shortening contributes to genomic instability, a hallmark of cancer that facilitates tumor formation and metastasis (5).

The non-high-density lipoprotein cholesterol to high-density lipoprotein cholesterol ratio (NHHR) is a recently developed composite indicator that provides a more nuanced understanding of lipid profiles (6). It reflects the balance between atherogenic and antiatherogenic lipoproteins and demonstrates a robust predictive value for adverse cardiovascular events (7). Recent studies have demonstrated that the NHHR is associated with an increased risk of cardiovascular disease and exhibits superior predictive capabilities for metabolic syndrome and insulin resistance compared with traditional lipid indicators such as total cholesterol and high-density lipoprotein cholesterol (HDL-C) levels (8, 9). Moreover, the NHHR has been demonstrated to outperform other composite indices, including the total cholesterol/HDL-C ratio, in predicting cardiovascular events, thereby establishing its broader clinical utility (10). Another comparative analysis with other lipid parameters also showed that the NHHR provided better value in predicting abdominal aortic aneurysms while maintaining diagnostic performance, especially in the elderly population (11). This finding contributes to the understanding of the relationship between NHHR and health status in the elderly population.

TL, a key biomarker of aging, has recently garnered increased attention due to its purported relationship with lipid metabolism. A Mendelian randomization study has provided preliminary evidence suggesting potential positive causal relationships between low-density lipoprotein cholesterol (LDL-C), very low-density lipoprotein cholesterol (VLDL-C), total cholesterol, and TL (12). These findings imply that alterations in lipid metabolism may influence TL dynamics, which could, in turn, affect the risk of developing age-related diseases. A cross-sectional study conducted among individuals in the US has further revealed a possible relationship between telomere length and HDL-C (13), thereby reinforcing the notion that lipid profiles are related to telomere dynamics.

Although mounting evidence links alterations in TL to alterations in lipid metabolism, limited information is available concerning the connection between NHHR and TL. Using data from the National Health and Nutrition Examination Survey (NHANES), this study aims to investigate the relationship between NHHR and TL and evaluate the NHHR as a practical biomarker for TL dynamics. By elucidating the association between NHHR and TL, this research aims to contribute to a deeper understanding of the influence of lipid metabolism on the aging process. The findings could have significant implications for clinical practice, particularly in the development of targeted interventions aimed at improving lipid profiles and preserving telomere length, which would ultimately enhance health span and reduce the burden of age-related diseases.

## 2 Materials and methods

### 2.1 Study population

The NHANES is a repeated cross-sectional survey that is being undertaken by the US National Center for Health Statistics (NCHS). It is a countrywide database that provides data on the health and nutritional status of adults and children in the US (14). The NCHS Ethics Review Board gave its approval to the NHANES study protocols. All research subjects executed formal informed consent documents.

**Figure 1:**
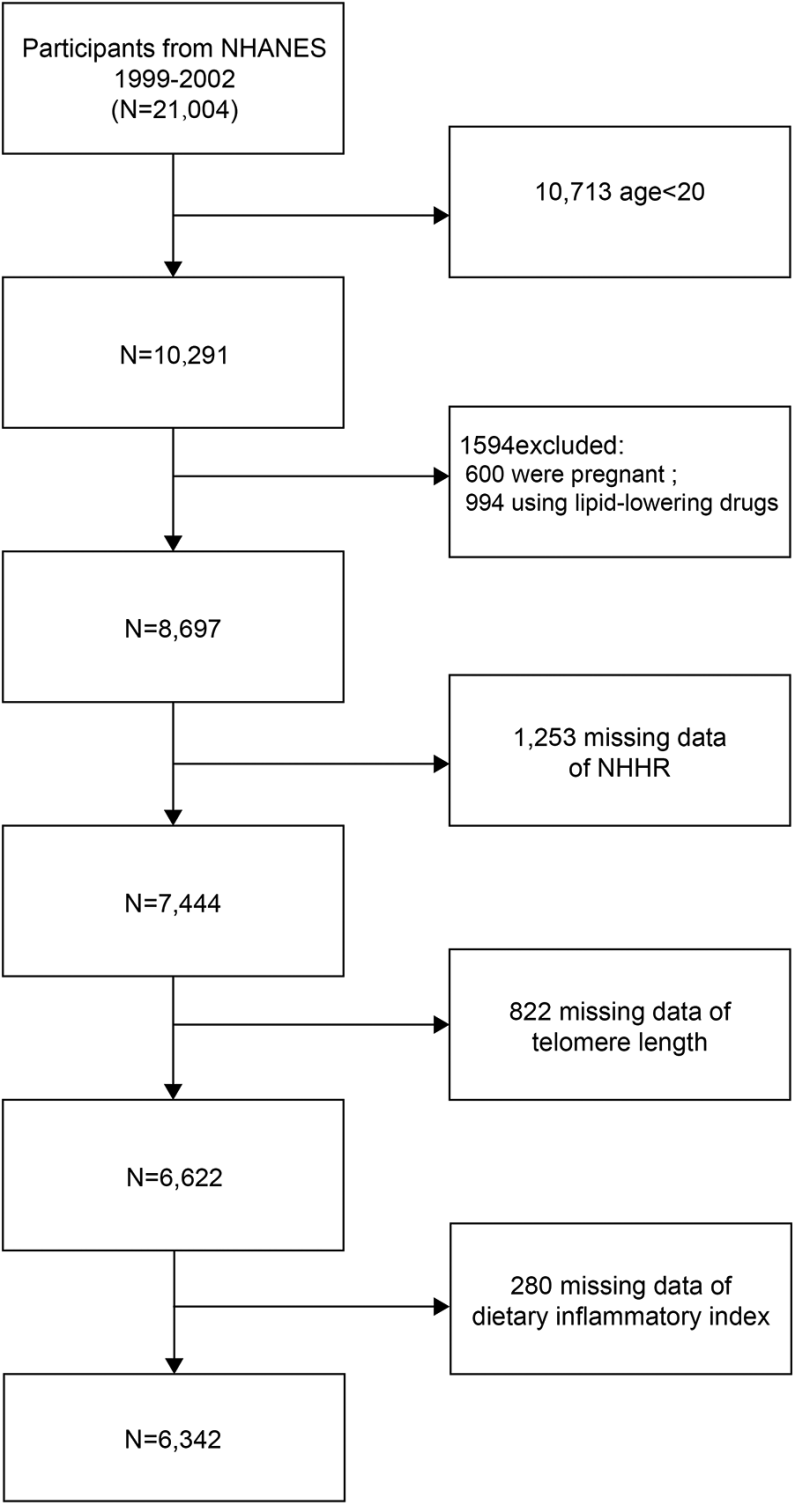
Flowchart of participant selection. NHANES, National Health and Nutrition Examination Survey; NHHR, non-high-density lipoprotein cholesterol to high-density lipoprotein cholesterol ratio.

This study performed a cross-sectional analysis based on the NHANES 1999–2002 cycles (1999–2000 and 2001–2002), as TL measurements were exclusively available during these cycles. Of the initial 21,004 participants, exclusions were made for individuals under 20 years of age (*n* = 10,713), pregnant women (*n* = 600), and those taking cholesterol-lowering medications (*n* = 994). Additional exclusions included participants with missing data for the NHHR (*n* = 1,253), telomere length (*n* = 822), and the dietary inflammatory index (DII) (*n* = 280). The final analytical cohort comprised 6,342 participants, as illustrated in Figure 1.

### 2.2 Study variables

The exposure variable was the NHHR, calculated by first subtracting HDL-C from TC to obtain non-HDL-C, and then dividing non-HDL-C by HDL-C (6).

The outcome variable was mean TL, determined by the ratio of TL-to-standard reference DNA (T/S ratio) in blood leukocytes from NHANES samples. Consistent with previous studies, the T/S ratio is referred to as TL in this analysis (15).

Covariates included both continuous and categorical variables. Continuous variables comprised age (years), body mass index (BMI), income-to-poverty ratio, and DII. The DII quantifies the inflammatory potential of dietary patterns, with higher scores reflecting more pro-inflammatory diets (16). For this analysis, DII scores were further classified as pro-inflammatory (DII > 0) or anti-inflammatory (DII < 0) (17). Categorical variables included gender, race, educational attainment, physical activity, smoking status, and medical history (diabetes, hypertension, and cancer). Given that TL was measured using DNA isolated from whole blood (18), hematological parameters were also included as covariates: white blood cell count (10^9^/L) and differential percentages (lymphocytes, monocytes, neutrophils, eosinophils, and basophils).

### 2.3 Statistical analysis

Statistical analysis was conducted using EmpowerStats (version 4.2) and R (version 4.2.0) software. The statistical analysis was conducted following the procedures recommended by the Centers for Disease Control and Prevention (CDC). To account for the complex survey design and non-response rates observed in the NHANES, sample weights were employed in the analyses. As the sample weights for the NHANES 1999–2000 and the NHANES 2000–2001 were derived from distinct US censuses, the Dietary Day one 4-Year sample weight provided by the NCHS was employed to account for the two reference populations (19). The continuous variable NHHR was transformed into a categorical variable based on tertiles. Both continuous and categorical variables were presented as means ± standard error (SE) or proportions, respectively, and were compared using t-tests with weights or weighted chi-square tests based on the various NHHR tertiles.

The relationship between NHHR and TL was investigated using multivariate linear regression analysis, with the beta coefficient (*β*) and 95% confidence interval (CI) calculated for each model. The study examined the non-linear relationship between TL and NHHR through the use of generalized additive model (GAM) regression analysis and smooth curve fitting. The variables that were categorized included smoking behavior, age, BMI, sex, educational attainment, dietary habits, hypertension, diabetes, and cancer. A two-sided *P*-value of less than 0.05 was considered statistically significant.

## 3 Results

### 3.1 Baseline characteristics

In this population-based study, after applying NHANES survey weights, the study population represented 149,541,747 US adults who were stratified into three NHHR tertiles (means ± SE: 1.88 ± 0.01, 3.05 ± 0.01, and 4.97 ± 0.04) (Table 1). An elevated NHHR was significantly associated with a shorter TL (T/S ratio: 1.09 ± 0.02–1.03 ± 0.02; *P* = 0.0005). A demographic analysis revealed that a higher NHHR correlated with increased age (43.83 ± 0.45–46.33 ± 0.43 years; *P* < 0.0001), higher male proportion (32.65%–66.19%; *P* < 0.0001), and decreased educational attainment (above high school: 59.15%–49.05%; *P* < 0.0001).

**Table 1:** Baseline characteristics of study participants according to NHHR tertiles.

Characteristics	Overall	Tertile 1	Tertile 2	Tertile 3	*P*-value
NHHR	3.30 ± 0.01	1.88 ± 0.01	3.05 ± 0.01	4.97 ± 0.04	<0.0001
Age (years)	45.16 ± 0.32	43.83 ± 0.45	45.32 ± 0.45	46.33 ± 0.43	0.0001
Income-to-poverty ratio	3.00 ± 0.05	3.13 ± 0.07	2.95 ± 0.08	2.93 ± 0.08	0.006
BMI (kg/m^2^)	27.94 ± 0.14	25.30 ± 0.20	28.35 ± 0.20	30.16 ± 0.17	<0.0001
WBC count(10^9^/L)	7.21 ± 0.06	6.88 ± 0.07	7.21 ± 0.07	7.54 ± 0.08	<0.0001
Lymphocyte percent (%)	30.02 ± 0.20	29.84 ± 0.23	30.03 ± 0.29	30.17 ± 0.27	0.4267
Monocyte percent (%)	8.10 ± 0.04	8.11 ± 0.07	8.18 ± 0.05	8.02 ± 0.06	0.1703
Neutrophils percent (%)	58.47 ± 0.21	58.75 ± 0.26	58.37 ± 0.28	58.28 ± 0.31	0.2547
Eosinophils percent (%)	2.79 ± 0.03	2.67 ± 0.05	2.80 ± 0.06	2.90 ± 0.04	0.0027
Basophils percent (%)	0.66 ± 0.01	0.67 ± 0.02	0.65 ± 0.01	0.67 ± 0.02	0.4461
TL, T/S ratio	1.06 ± 0.02	1.09 ± 0.02	1.06 ± 0.02	1.03 ± 0.02	0.0005
DII	1.08 ± 0.07	0.99 ± 0.06	1.07 ± 0.08	1.19 ± 0.08	0.0415
Gender (%)					<0.0001
Male	49.21	32.65	48.55	66.19	
Female	50.79	67.35	51.45	33.81	
Race/ethnicity (%)					<0.0001
Mexican American	7.25	6.29	8.42	7.13	
Other Hispanic	6.46	4.95	6.91	7.53	
Non-Hispanic White	72.94	73.41	70.14	75.04	
Non-Hispanic Black	9.53	11.94	10.66	6.1	
Other races	3.82	3.4	3.86	4.21	
Education level (%)					<0.0001
Less than high school	20.72	17.88	22.3	22.08	
High school or GED	25.89	22.97	25.79	28.87	
Above high school	53.39	59.15	51.9	49.05	
Physical activity (%)					0.1193
None	20.62	19.46	19.73	22.59	
Low	27.14	24.72	27.99	28.73	
Medium	18.98	19.75	19.93	17.35	
High	33.25	36.06	32.35	31.32	
Smoking status (%)					0.0304
Current	42.58	41.99	41.48	43.97	
Former	8.38	11.11	6.3	7.84	
Never	49.04	46.91	52.22	48.18	
Hypertension (%)					<0.0001
Yes	23.44	18.81	25.96	25.71	
No	76.56	81.19	74.04	74.29	
Diabetes (%)					<0.0001
Yes	5.42	3.38	5.71	7.17	
No	93.63	96.06	93.37	91.48	
Borderline	0.95	0.56	0.92	1.35	
Cancer history (%)					0.07
Yes	7.79	8.67	8.23	6.52	
No	92.21	91.33	91.77	93.48	

Abbreviations: NHHR, non-high-density lipoprotein cholesterol to high-density lipoprotein cholesterol ratio; BMI, body mass index; WBC, white blood cell; DII, dietary inflammatory index; TL, telomere length; T/S ratio, telomere length-to-standard reference DNA ratio; GED, general educational development.

Data are presented as mean ± standard error for continuous variables and percentages for categorical variables.

Subjects with an elevated NHHR demonstrated significantly increased inflammatory markers, including higher white blood cell counts (6.88 ± 0.07–7.54 ± 0.08 × 10^9^/L; *P* < 0.0001) and DII scores (0.99 ± 0.06–1.19 ± 0.08; *P* = 0.0415). In addition, these subjects exhibited an increased BMI (25.30 ± 0.20–30.16 ± 0.17 kg/m^2^; *P* < 0.0001) and a higher prevalence of hypertension (18.81%–25.71%; *P* < 0.0001) and diabetes (3.38%–7.17%; *P* < 0.0001). These findings suggest that elevated NHHR is independently associated with adverse demographic, inflammatory, and cardiometabolic profiles in the US adult population.

### 3.2 Association between NHHR and TL

The unadjusted Model 1 demonstrated a statistically significant inverse correlation between NHHR and TL (*β* = −0.02, 95% CI: −0.02 to −0.01, *P* < 0.0001) (Table 2). This association remained significant after controlling for basic demographic variables in Model 2 (*β* = −0.01, 95% CI: −0.02 to −0.01, *P* = 0.0015). However, in the fully adjusted Model 3, the association was found to be attenuated and became non-significant (*β* = −0.005, 95% CI: −0.015 to 0.004, *P* = 0.4030).

**Table 2:** Association between NHHR and TL.

Exposure	Model 1 [*β* (95% CI), *P*-value]	Model 2 [*β* (95% CI), *P*-value]	Model 3 [*β* (95% CI), *P*-value]
Continuous NHHR	−0.02 (−0.02 to −0.01),<0.0001	−0.01 (−0.02 to −0.01), 0.0015	−0.005 (−0.015 to 0.004), 0.4030
NHHR group			
Tertile 1	Reference	Reference	Reference
Tertile 2	−0.03 (−0.05 to −0.01), 0.0166	−0.01 (−0.04 to 0.01), 0.2185	−0.02 (−0.05 to 0.02), 0.4903
Tertile 3	−0.06 (−0.08 to −0.03), 0.0001	−0.04 (−0.06 to −0.01), 0.0126	−0.02 (−0.07 to 0.03), 0.5620
*P* for trend	0.0001	0.0123	0.4643

95% CI, 95% confidence interval.

Model 1, no covariates were adjusted. Model 2, age, gender, and race were adjusted. Model 3, age, gender, race, education level, BMI, income-to-poverty ratio, smoking behavior, diabetes, hypertension, physical activity, DII, white blood cell count, lymphocyte percent, monocyte percent, segmented neutrophils percent, eosinophils percent, and basophils percent were adjusted.

**Figure 2:**
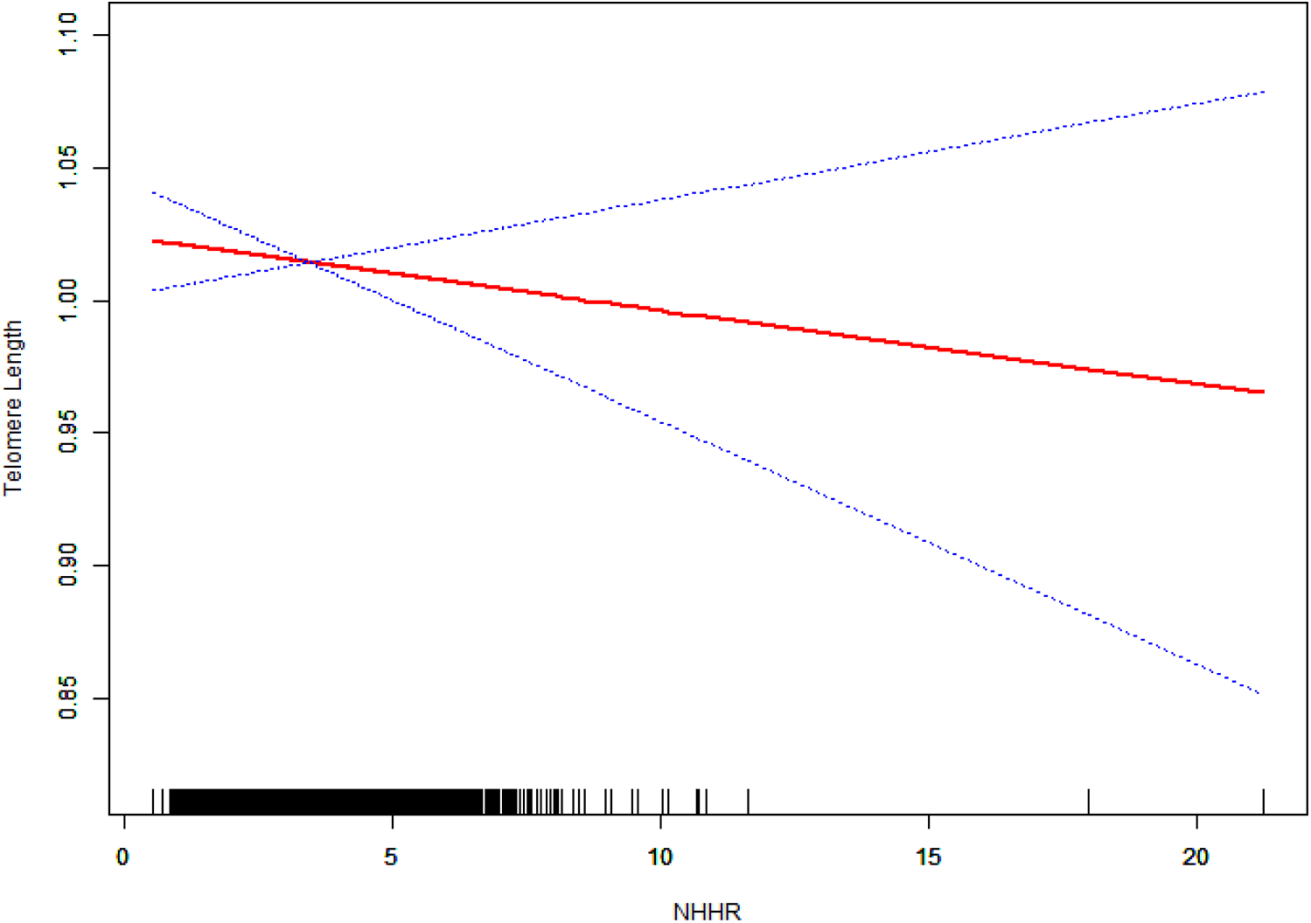
The linear relationship between NHHR and TL. The solid red line represents the fitted values of TL across NHHR values, and the blue dotted lines represent the 95% confidence intervals. The rug plot at the bottom shows the distribution of NHHR values in the study population. The model was adjusted for potential confounders in Model 3.

In analyses based on tertiles, the highest NHHR tertile exhibited significant negative associations in both Model 1 (*β* = −0.06, 95% CI: −0.08 to −0.03, *P* = 0.0001) and Model 2 (*β* = −0.04, 95% CI: −0.06 to −0.01, *P* = 0.0126). However, this association was not significant in Model 3 (*β* = −0.02, 95% CI: −0.07 to 0.03, *P* = 0.5620). The results of the trend analyses indicated the presence of a significant dose–response relationship in Models 1 and 2 (*P* for trend = 0.0001 and 0.0123, respectively), which, however, was no longer statistically significant in Model 3 (*P* for trend = 0.4643). The results of the smoothing curve fitting revealed a significant linear inverse association between NHHR and TL (*P* < 0.05). Specifically, as NHHR values increased, a consistent decrease in TL was observed (Figure 2).

### 3.3 Subgroup analyses

**Figure 3:**
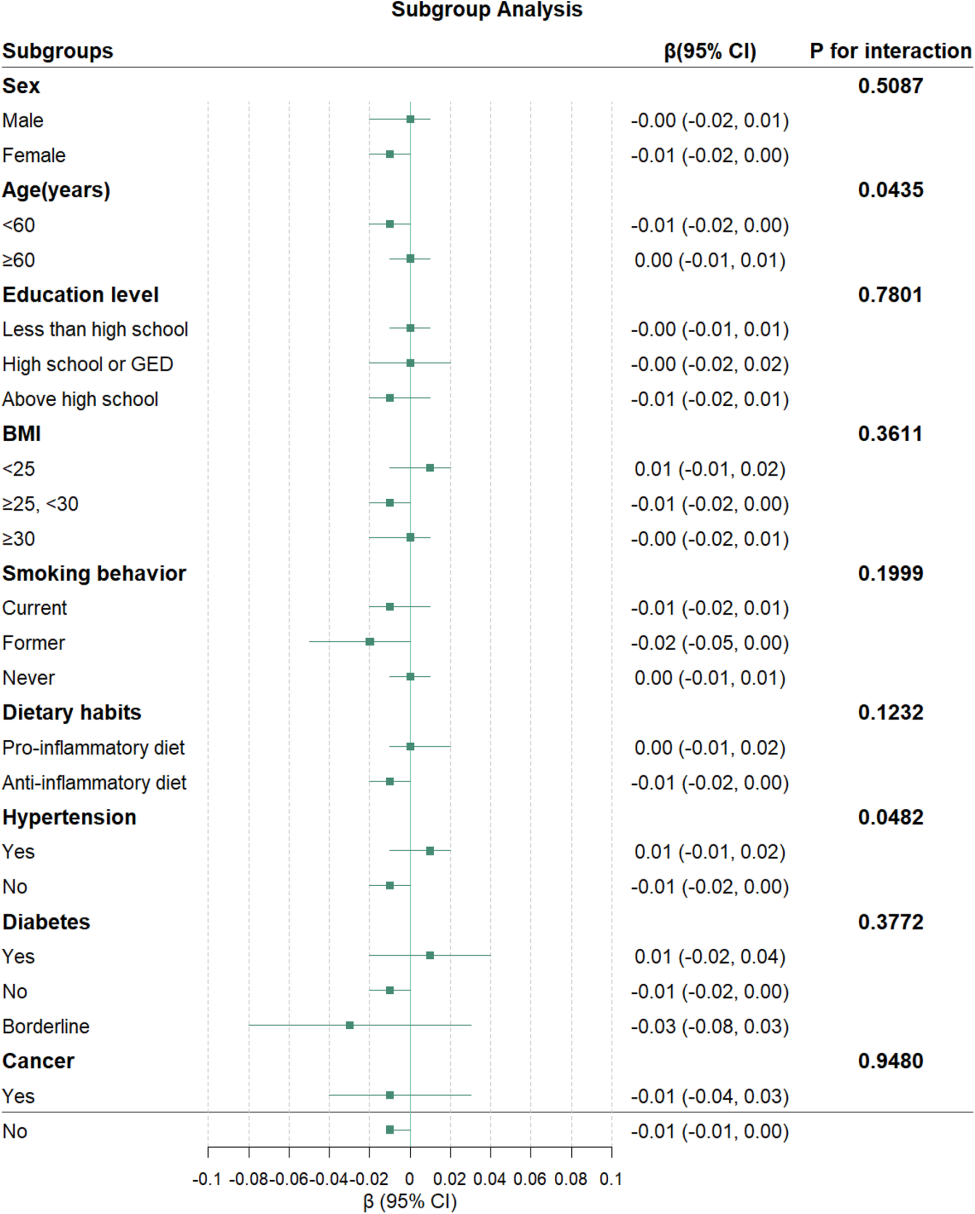
Association between NHHR and TL in subgroups. The green squares represent the *β* coefficients, and the horizontal lines indicate 95% CI for each subgroup. The model was adjusted for potential confounders in Model 3, except for the stratification factor in each subgroup.

Subgroup analyses revealed significant interactions for age (*P* for interaction = 0.0435) and hypertension status (*P* for interaction = 0.0482) in the association between NHHR and TL (Figure 3). No significant interactions were observed for sex, education level, BMI, smoking behavior, diabetes status, cancer history, or dietary habits (all *P* for interaction > 0.05).

### 3.4 Differential associations of NHHR with TL by age and hypertension status

**Figure 4:**
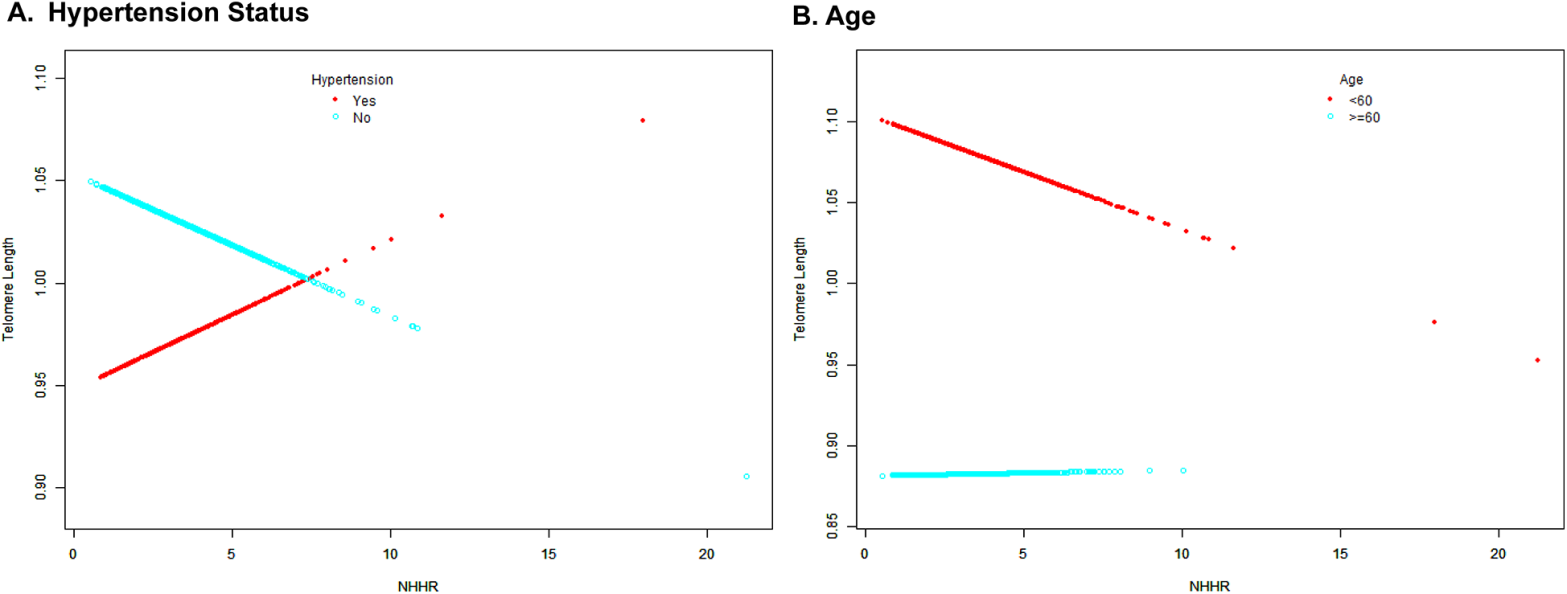
Smooth curve fitting for the association between NHHR and TL stratified by age **(A)** and hypertension status **(B)**. The colors of the dots and fitted curves are as follows: red is used for the hypertensive/age <60 years groups, while light blue is used for the non-hypertensive/age ≥60 years groups. The vertical axis represents telomere length, while the horizontal axis represents NHHR values.

Further analyses were conducted to identify significant interaction factors identified in subgroup analyses. The results of the smooth curve fitting revealed distinct patterns across different stratification factors (Figure 4). Concerning hypertension status, non-hypertensive participants exhibited a consistent negative trend, whereas hypertensive participants demonstrated a positive correlation. With regard to age stratification, participants aged less than 60 years exhibited a steeper negative slope in comparison with those aged 60 years or older, who demonstrated a relatively stable trend.

## 4 Discussion

This study identified a significant inverse association between NHHR and TL in a nationally representative sample of US adults for the first time. Participants in the highest NHHR tertile, compared with those in the lowest tertile, were older, had a higher proportion of males, and demonstrated more adverse inflammatory and cardiometabolic profiles. Specifically, individuals in the highest tertile exhibited elevated white blood cell counts, higher DII scores, increased BMI, and a greater prevalence of hypertension and diabetes. These findings suggest that the NHHR may serve as an indicator not only of lipid metabolism but also of systemic inflammation and metabolic health, both of which are potential contributors to telomere shortening.

Previous studies have demonstrated the association between adverse lipid profiles and cellular aging. For example, a shorter TL was closely linked to a higher triglyceride-glucose index in a recent observational study involving 6,489 non-diabetic individuals in the United States (20). The Fels Longitudinal Study also reported that apolipoprotein B concentrations and higher total cholesterol/HDL-C ratios were independently associated with shorter telomeres (21). Similarly, an inverse association between TL and changes in total blood lipid concentrations was observed in a male population in Tehran (22), reinforcing the critical role of lipid metabolism in telomere dynamics. The NHHR has emerged as a significant biomarker for evaluating cardiovascular risk and its association with aging-related diseases (23–25). Compared with traditional lipid indicators, the NHHR provides a more comprehensive assessment by integrating both non-HDL-C and HDL-C levels, thus accounting for the protective effects of HDL-C (26, 27). Notably, our study revealed an inverse relationship between NHHR and TL, further supporting the link between lipid profiles and cellular aging.

The relationship between NHHR and TL can be explained through several interconnected mechanisms, including oxidative stress, lipid metabolism, inflammation, and telomerase activity. A prevailing hypothesis suggests that lipid metabolism is closely tied to oxidative stress levels (28). Oxidative stress is not only a key contributor to aging and age-related chronic diseases, such as cardiovascular disease, but also accelerates telomere attrition (29, 30). Elevated NHHR reflects a lipid imbalance characterized by decreased HDL-C and increased non-HDL-C levels. This imbalance can exacerbate oxidative stress and inflammatory responses, both of which accelerate telomere shortening. Non-HDL-C primarily comprises VLDL-C, intermediate-density lipoprotein cholesterol (IDL-C), and LDL-C (31). The clearance of LDL-C is regulated by hepatic LDL receptors (LDLRs), which remove approximately 75% of circulating cholesterol via endocytosis (32). Non-HDL cholesterol is eliminated by the LDLR through the recognition of apolipoprotein B100 (Apo B100) and apolipoprotein E (Apo E) on lipoproteins. Recent studies in aging mice have revealed that hepatic mitochondrial function declines with age, leading to increased reactive oxygen species (ROS) production and oxidative stress. This, in turn, promotes glucose uptake and glycolysis, ultimately resulting in cholesterol accumulation in the liver (33). Furthermore, it has been suggested that when cholesterol levels in the endoplasmic reticulum of hepatocytes exceed 5% of total blood lipids, LDLR mRNA transcription is inhibited, reducing LDLR expression and attenuating LDL-C clearance, thereby increasing circulating non-HDL-C levels (34). Previous research has also established serum cholesterol as a significant contributor to oxidative stress (35). A randomized trial demonstrated that persistently elevated cholesterol levels can trigger inflammatory responses and promote ROS generation and accumulation, which damages DNA through oxidative stress (36). Oxidative stress and inflammation may also enhance leukocyte turnover, reducing TL with each cell cycle (37).

In addition to its role in clearing lipid deposits from arterial walls, HDL also exhibits antioxidant, anti-inflammatory, and antiproteolytic properties during aging (38, 39). Seo et al. found that HDL-C levels significantly declined with age in mice of different ages (33). HDL has been proposed to participate in stress-related signaling pathways (40). In plasma and interstitial cells, HDL reduces oxidative stress, cytotoxicity, and cellular damage (41). Declining HDL-C levels with age may impair the regulation of oxidative stress and increase cellular damage, thereby accelerating leukocyte renewal and telomere shortening. Notably, prior research demonstrated that the HDL-associated enzyme paraoxonase 1 (PON1), which contributes to the antioxidant activity of HDL, loses its protective effect with age. This suggests that telomere shortening may also be linked to the deterioration of HDL function during aging (42). In summary, elevated NHHR may create a vicious cycle of increased oxidative stress and elevated cholesterol levels, which can directly or indirectly damage telomeres.

Telomerase is an enzyme that counteracts telomere shortening during cell division by adding telomeric repeats to chromosome ends (1). Reduced telomerase activity accelerates telomere attrition, contributing to cellular aging and the development of age-related diseases. Oxidative stress not only directly damages telomeric DNA but also inhibits telomerase activity, further exacerbating telomere shortening (43, 44). Although our study did not directly measure telomerase activity, elevated NHHR may influence telomerase function through increased oxidative stress and inflammation. Dyslipidemia has been associated with reduced telomerase expression and activity in endothelial cells (45). In addition, pro-inflammatory cytokines have been shown to downregulate telomerase activity (46). These mechanisms suggest that the NHHR may affect TL by modulating telomerase function, further linking lipid metabolism to cellular aging processes.

In the fully adjusted Model 3, which accounted for covariates such as physical activity and the DII, the negative association between NHHR and TL became non-significant. This attenuation may reflect the influence of lifestyle factors on telomere dynamics. Physical activity and diet are well-established factors that impact both lipid metabolism and telomere maintenance. Higher levels of physical activity have been associated with longer telomeres, potentially due to reductions in oxidative stress and inflammation, as well as improvements in lipid profiles (47). Regular exercise enhances antioxidant defenses and mitigates the oxidative stress that contributes to telomere shortening. Conversely, sedentary behavior may accelerate telomere attrition through increased oxidative stress and reduced telomerase activity (48, 49). Dietary patterns also play a critical role in telomere biology (50). The DII quantifies the inflammatory potential of the diet of an individual, with higher scores indicating a pro-inflammatory diet. Diets rich in pro-inflammatory foods have been linked to increased oxidative stress and shorter TL (51). In our study, participants with a higher NHHR tended to have higher DII scores and lower levels of physical activity. These factors may confound the relationship between NHHR and TL, as both physical inactivity and pro-inflammatory diets independently contribute to telomere shortening. The inclusion of physical activity and the DII in Model 3 may have attenuated the NHHR-TL association by accounting for these lifestyle factors. This suggests that physical activity and dietary patterns may modulate the impact of the NHHR on telomere length, potentially through mechanisms involving reduced oxidative stress and improved lipid metabolism. These findings highlight the importance of considering lifestyle behaviors when evaluating the relationship between lipid profiles and cellular aging markers.

Our subgroup analyses revealed nuanced interactions between NHHR and TL across different populations. In non-hypertensive individuals, the negative correlation between NHHR and TL remained significant, supporting the hypothesis that adverse lipid profiles contribute to telomere shortening via oxidative stress and inflammation. However, in hypertensive participants, this association was attenuated and even showed a positive trend. This could be attributed to the confounding effects of antihypertensive medications, which may alter lipid metabolism and influence telomere dynamics (52). Age-stratified analyses showed that the negative association between NHHR and TL was more pronounced in participants under 60 years of age compared with those 60 and older. This may be due to age-related changes in lipid metabolism and lifestyle factors that modify the relationship between NHHR and TL. In older adults, cumulative exposure to adverse lipid profiles may reach a threshold where additional increases in the NHHR have a diminished impact on TL. In addition, survival bias may play a role, as individuals with healthier lipid profiles and more robust telomere maintenance mechanisms are more likely to survive to advanced ages (53).

In conclusion, this study highlights the potential of the NHHR as a biomarker for biological aging, linking lipid metabolism, systemic inflammation, and telomere dynamics. These findings underscore the importance of considering lifestyle factors, such as physical activity and diet, when evaluating the relationship between lipid profiles and cellular aging. Further longitudinal and mechanistic studies are warranted to validate these findings and elucidate the underlying pathways.

This study has several notable strengths. First, it is the first to demonstrate an association between NHHR and TL among US adults. It identified a linear correlation between NHHR and TL, providing new insights into this relationship. Second, this study focused on the NHHR metric, which has proven to be an excellent predictive marker across a wide range of diseases. This focus enhances the clinical relevance and significance of the findings compared with earlier studies of a similar nature. Lastly, this study utilized data from the NHANES, which employs a complex multistage probability sampling methodology. This approach allows the results to be generalized to other comparable populations during the study period. In addition, the large sample size enabled the study to account for numerous potential covariates that could influence NHHR variations and TL alterations, increasing the robustness of the findings.

However, this study also has several limitations. First, due to its cross-sectional design, it was able to present only observational evidence of the correlation between NHHR and TL, and it could not establish causality. The cross-sectional nature of TL measurements also fails to capture dynamic changes in telomere attrition rates. Future prospective and longitudinal studies are needed to address this limitation. Second, this study excluded individuals who were pregnant or taking lipid-lowering medications, as these factors significantly influence TL and cholesterol levels (54). Consequently, the findings do not apply to these groups. Third, all participants in this study were drawn from the US general population, which raises questions about the applicability of the findings to populations in other countries or regions. Further research in diverse populations is necessary to confirm the generalizability of these results. Furthermore, the lack of direct measurements of telomerase activity restricts our understanding of the mechanistic pathways involved. Future longitudinal studies are warranted to explore the causal relationships and assess the impact of interventions targeting physical activity, diet, and lipid profiles on telomere dynamics. Investigating telomerase activity with the NHHR could provide deeper insights into the mechanisms linking lipid metabolism to cellular aging. Such research may inform strategies aimed at preserving telomere length and promoting healthy aging.

## 5 Conclusion

This study revealed a significant linear inverse association between NHHR levels and telomere length in the US population for the first time. The NHHR, obtained from routine clinical testing, shows potential as a novel biomarker for TL prediction. During standard lipid monitoring, elevated NHHR levels may indicate accelerated biological aging. This provides a practical screening approach for age-related conditions. Future prospective studies are needed to validate the predictive value of the NHHR.

## Data Availability

The original contributions presented in the study are included in the article/Supplementary Material, and further inquiries can be directed to the corresponding author.
